# Phase I safety trial of intravenous ascorbic acid in patients with severe sepsis

**DOI:** 10.1186/1479-5876-12-32

**Published:** 2014-01-31

**Authors:** Alpha A Fowler, Aamer A Syed, Shelley Knowlson, Robin Sculthorpe, Don Farthing, Christine DeWilde, Christine A Farthing, Terri L Larus, Erika Martin, Donald F Brophy, Seema Gupta, Bernard J Fisher, Ramesh Natarajan

**Affiliations:** 1 Division of Pulmonary Disease and Critical Care Medicine, Department of Internal Medicine, School of Medicine, Virginia Commonwealth University, PO Box 980050, Richmond, VA 23298-0050, USA; 2 Department of Critical Care Nursing, Virginia Commonwealth University Health System, Richmond, Virginia, USA; 3 Investigational Drug Services, Department of Pharmacy Services, School of Pharmacy, Virginia Commonwealth University, Richmond, Virginia, USA; 4 Division of Nephrology, Department of Internal Medicine, School of Medicine, Virginia Commonwealth University, Richmond, Virginia, USA; 5 Department of Pharmacotherapy & Outcomes Science, School of Pharmacy, Virginia Commonwealth University, Richmond, Virginia, USA; 6 Health Diagnostic Laboratory, Richmond, Virginia, USA

**Keywords:** Ascorbic acid, Biological markers, Clinical trials phase I as topic, Multiple organ failure, Organ dysfunction scores, Sepsis

## Abstract

**Background:**

Parenterally administered ascorbic acid modulates sepsis-induced inflammation and coagulation in experimental animal models. The objective of this randomized, double-blind, placebo-controlled, phase I trial was to determine the safety of intravenously infused ascorbic acid in patients with severe sepsis.

**Methods:**

Twenty-four patients with severe sepsis in the medical intensive care unit were randomized 1:1:1 to receive intravenous infusions every six hours for four days of ascorbic acid: *Lo-AscA* (50 mg/kg/24 h, n = 8), or *Hi-AscA* (200 mg/kg/24 h, n = 8), or *Placebo* (5% dextrose/water, n = 8). The primary end points were ascorbic acid safety and tolerability, assessed as treatment-related adverse-event frequency and severity. Patients were monitored for worsened arterial hypotension, tachycardia, hypernatremia, and nausea or vomiting. In addition Sequential Organ Failure Assessment (SOFA) scores and plasma levels of ascorbic acid, C-reactive protein, procalcitonin, and thrombomodulin were monitored.

**Results:**

Mean plasma ascorbic acid levels at entry for the entire cohort were 17.9 ± 2.4 μM (normal range 50-70 μM). Ascorbic acid infusion rapidly and significantly increased plasma ascorbic acid levels. No adverse safety events were observed in ascorbic acid-infused patients. Patients receiving ascorbic acid exhibited prompt reductions in SOFA scores while placebo patients exhibited no such reduction. Ascorbic acid significantly reduced the proinflammatory biomarkers C-reactive protein and procalcitonin. Unlike placebo patients, thrombomodulin in ascorbic acid infused patients exhibited no significant rise, suggesting attenuation of vascular endothelial injury.

**Conclusions:**

Intravenous ascorbic acid infusion was safe and well tolerated in this study and may positively impact the extent of multiple organ failure and biomarkers of inflammation and endothelial injury.

**Trial registration:**

ClinicalTrials.gov identifier NCT01434121.

## Background

The incidence of sepsis and sepsis-associated organ failure continues to rise in Intensive Care Units worldwide with studies from multiple countries showing that organ failure contributes cumulatively to patient mortality [1-3]. Patients with severe sepsis suffer higher mortality rates compared to patients with organ failure but no sepsis. Despite over 15,000 patients studied and over 1 billion dollars in study costs effective sepsis therapy remains elusive [4,5]. Clinical trials that have targeted mediators of inflammation or coagulation such as atorvastatin [6] or activated protein C [7] have not reduced septic mortality, suggesting that single-target therapy fails to meet the challenges of complex multicellular activation and interactions.

Recent studies suggest that ascorbic acid may attenuate pathological responses in septic microvasculature. Armour et al. and Wu et al. showed that ascorbic acid infusion improved capillary blood flow, microvascular barrier function, and arteriolar responsiveness to vasoconstrictors in septic animals [8,9]. Recently, we showed that parenterally infusing ascorbic acid at a concentration of 200 mg/kg attenuated vascular lung injury in septic mice by multiple mechanisms, including attenuation of the proinflammatory mediators, enhanced alveolar epithelial barrier function, increased alveolar fluid clearance, and prevention of sepsis-induced coagulopathy [10,11]. In addition, ascorbic acid deficient mice were found to be more susceptible to sepsis-induced multiple organ dysfunction and parenteral infusion of ascorbic acid attenuated the injury (lung, kidney, liver) [12].

Subnormal plasma ascorbic acid concentrations in septic patients correlate inversely with the incidence of multiple organ failure and directly with survival [13]. Ascorbic acid depletion in sepsis results from: 1) ascorbic acid consumption by reduction of plasma free iron, 2) ascorbic acid consumption by the scavenging of aqueous free radicals, and 3) by destruction of the oxidized form of ascorbic acid, dehydroascorbic acid [14]. Dosing and bio-distribution data in humans show that pharmacological concentrations of ascorbic acid can only be attained following intravenous administration [15]. Surprisingly, few studies in critically ill patients infusing ascorbic acid have been performed. Nathens and colleagues infused ascorbic acid at 1 gram every 8 hours combined with oral vitamin E for 28 days in 594 surgically critically ill patients and found a significantly lower incidence of acute lung injury and multiple organ failure [16]. Tanaka et al. infused ascorbic acid continuously at 66 mg/kg/hour for the first 24 hours in patients with greater than 50% surface area burns and showed significantly reduced burn capillary permeability [17]. A single report (published as abstract only) of a clinical study of large intravenous doses of ascorbic acid, and other antioxidants (tocopherol, N-acetyl-cysteine, selenium), in patients with established ARDS showed a 50% reduction in mortality [18]. Clinical protocols currently in use for hospitalized septic patients fail to normalize ascorbic acid levels. Ascorbic acid dosages utilized in this trial arose from our preclinical work.

In the current trial, we sought to determine whether intravenous ascorbic acid was safe to administer to critically ill patients with severe sepsis and to determine if ascorbic acid had an impact on organ failure and a priori selected blood biomarkers. We measured C-reactive protein and procalcitonin as systemic markers of inflammation while choosing thrombomodulin as a marker of vascular injury [19-21]. The work reported in this study has previously been presented at the American Thoracic Society International Meeting [22].

## Methods

This study was approved by the VCU Institutional Review Board (IRB). The IRB approval number assigned to this trial was: HM12903. The trial was conducted under a randomized double blind placebo-controlled format. A multi-departmental data safety monitoring board oversaw the trial.

### Patient enrollment

Patients were screened and enrolled following admission to the Medical Respiratory Intensive Care Unit in the VCU Medical Center, Richmond, Virginia. Severe sepsis was defined as: 1) *Presence of a systemic inflammatory response*: (fever: >38°C or hypothermia: <36°C (core temp only), heart rate > 90 beats/min, leukocytosis: >12,000 WBC/μL or leukopenia: <4,000 WBC/μL or >10% band forms) [23], 2) *Suspected or proven infection*, and 3) *Presence of sepsis-induced organ dysfunction*: Arterial hypoxemia (P_a_O_2_/F_i_O_2_ < 300), systolic blood pressure (SBP) < 90 mm Hg or SBP decrease > 40 mm Hg unexplained by other causes, Lactate > 2.5 mMol/L Urine output < 0.5 ml/kg/hour for greater than two hours despite fluid resuscitation, platelet count < 100,000, acutely developing coagulopathy (INR > 1.5), Bilirubin > 2 mg/dL. If these three criteria were met within 48 hours of ICU admission, informed consent was obtained from family members of patients deemed eligible for the study. Study groups in this trial were 1) *Placebo*: 5% dextrose and water; 2) *Low dose ascorbic acid (Lo-AscA)*: 50 mg/kg/24 hours; or 3) *High dose ascorbic acid (Hi-AscA)*: 200 mg/kg/24 hours. Ascorbic acid dosage was divided into 4 equal doses and administered over 30 minutes every 6 hours for 96 hours in 50 ml of 5% dextrose and water. Study drug infusion was initiated 2 to 4 hours following informed consent and randomization.

The study blind was established and maintained by the VCU Investigational Pharmacy Department where the study drug was prepared, hooded, and dispensed. Subjects were assigned to one of three dosing groups (0 mg/kg/day, 50 mg/kg/day, or 200 mg/kg/day) in a 1:1:1 ratio using a randomization scheme generated by using *Research Randomizer*[24]. Placebo or study drug was prepared in 50 mL polyvinyl chloride intravenous infusion bags (Viaflex, Baxter Healthcare, Deerfield, IL). Ascorbic Acid Injection, USP, (Bioniche Pharma, Lake Forest, IL) was used. Ascorbic acid or placebo solutions were prepared in matching volumes with amber shrouding for light protection and to preserve the blind. Air was removed from IV bags for protection against ascorbic acid oxidation. Ascorbic acid was stored at 2–8°C for up to 24 hours prior to use. Preliminary experiments showed no oxidation under these brief storage conditions.

### Study data management

Collected data was managed using REDCap (Research Electronic Data Capture), a secure, web-based data collection and storage tool hosted at VCU [25].

### Assessment of organ failure

Organ failure was assessed using the *Sequential Organ Failure Assessment* (SOFA) score described by Vincent and colleagues [26]. Scores were calculated at enrollment and at 24, 48, 72, and 96 hours given the predictive value of serial SOFA scores reported by Ferreira et al. [27]. Laboratory data and physiologic measures for calculating SOFA scores were monitored daily and recorded into REDCap. Data was normalized using the delta total SOFA score (total maximum SOFA score at study entry minus total maximum SOFA score over the 4-day study period) [28,29].

### Study drug infusion and safety monitoring

Vital signs were monitored every 5 minutes during infusion and every 5 minutes for 45 minutes afterwards by bedside Medical Respiratory Intensive Care Unit (MRICU) Nursing and the investigative team. Patient safety in this Phase I trial was paramount. Four objective indices were monitored during and after ascorbic acid infusion: 1) Hypotension: Defined as a fall in mean arterial blood pressure of 20 mm Hg during or following infusion, 2) Tachycardia: Defined as an increase in heart rate of 20 beats per minute during or following infusion, 3) Hypernatremia: Standard of care utilizes 0.9% saline for volume resuscitation. L-Ascorbic acid preparation used for this study presented a minor sodium load, therefore a potential for hypernatremia to develop existed and 4) Nausea or vomiting: were monitored both during and after ascorbic acid administration by investigators and by MRICU nursing staff. If one of the adverse events listed above was observed, ICU nursing was equipped with bedside algorithms designed to manage the adverse event. If an event was observed, drug infusion was halted. If the event resolved, drug infusion was restarted at 50% of the original infusion rate. If the event recurred, the patient was removed from the study. If no adverse event was observed, patients were infused for 4 days. Patients were then followed clinically for 28 days.

### Blood samples

Whole venous blood was drawn into sterile Vacutainer® tubes (Becton, Dickinson & Co., Franklin Lakes, NJ): serum tube (BD 367812, red top, clot activator) and plasma tube (lavender top, BD 367861, K2EDTA). Serum samples were allowed to coagulate for 60 min at room temperature. Plasma and serum were separated by centrifugation. An aliquot of freshly isolated plasma was processed for ascorbic acid analysis. Remaining plasma and serum were aliquoted and frozen at -70°C until assayed.

### Plasma ascorbic acid measurement

*Plasma Ascorbic Acid Stability*: Preliminary work optimized conditions for stabilizing ascorbic acid in EDTA plasma samples. Briefly, 0.4 ml of cold 20% trichloroacetic acid (TCA) and 0.4 ml of cold 0.2% dithiothreitol (DTT) were added to 0.2 ml of plasma, vortexed for 2 min, and centrifuged (10,000 g, 10 min, 4°C). Supernatants were aliquoted and frozen at -70°C for batch analysis. Quality control samples consisted of normal plasma spiked with ascorbic acid (100 & 1,000 μM), processed in the same manner, and stored with patient samples. *Plasma Ascorbic Acid Concentrations*: Plasma ascorbic acid levels were quantified in all patients at enrollment then ***just prior to administration*** of the 12, 24, 36, 48, 72, and 96 hour ascorbic acid dosing. Concentrations were measured using high pressure liquid chromatography (HPLC) with UV detection. Chromatography was performed on an Onyx Monolithic C18 Column (100 × 4.6 mm; Phenomenex, Torrance, CA) with a mobile phase using a gradient buffer (dipotassium phosphate), ion pairing reagent (tretrabutyl amonium chloride), and acetonitrile at a flow rate 0.8 ml/min. Detection was at 265 nm and ascorbic acid levels quantified using peak area analysis and external standardization. Ascorbic acid standards (0–1,000 μM) were freshly prepared and treated in the same way as the test plasma samples.

### Biomarkers

Biomarkers measured for this study were identified prior to the start of the study. *C-Reactive Protein (CRP)*: A high sensitivity C-reactive protein (hsCRP) assay was performed in collaboration with Health Diagnostics Laboratories, Richmond, Virginia using the Roche hsCRP kit (catalog # 11972855216) on a Roche automated chemistry analyzer. *Procalcitonin (PCT)*: Procalcitonin levels were quantified using a sandwich ELISA kit according to manufacturer’s instructions (RayBiotech, Inc., Norcross, GA). *Thrombomodulin (TM)*: Plasma levels were quantified using an enzyme-linked immunosorbent assay kit (IMUBIND; American Diagnostica Inc., Stamford, Connecticut, USA). Samples were incubated in microwells precoated with a monoclonal antibody specific for human thrombomodulin.

See Additional file 1 for description of methods utilized for biomarker analysis.

### Statistical analysis

All analyses in this study were pre-specified. Statistical analysis was performed using SAS 9.3 and Graphpad PRISM 6.0. The results are expressed as means ± SE. Differences between and within groups were analyzed using two-factor analysis of variance with Tukey’s studentized range test. Summary data is reported as mean ± SEM. Statistical significance was confirmed at a p value of <0.05. Organ dysfunction analysis was based on the evolution (slopes) of the delta daily total SOFA score (change in daily total SOFA score compared with day 0) over 4 study days by comparing the regression coefficients using Student’s t-test [28,29].

## Results

### Enrolled study patients

Over a one year period, 35 patients were screened and 26 patients were enrolled. Reasons for excluding the 9 patients are as follows: a) 3 patients had terminal cancer and were not expected to survive for 24 hours; b) Informed consent could not be obtained in two homeless septic patients, and c) family members refused consent in 4 patients. Eight were enrolled in the placebo group, 8 enrolled in the Lo-AscA group, and 10 enrolled in the Hi-AscA. One patient in the Hi-AscA group was withdrawn by family members and transferred to another institution. One other Hi-AscA patient was withdrawn after Hemophagocytic Syndrome plus sepsis was recognized. These two patients are not included in the analysis. All patients received full ICU standard of care support. Table 1 shows the demographics of enrolled patients. The APACHE II and SOFA scores between groups were statistically identical. Table 2 indicates the underlying diagnosis for patients entered into the trial, the organ system affected, the source and identification of organisms in the patients, and on day one of entry into the trial whether acute kidney injury or respiratory failure was present. Secondary outcomes (i.e., days on vasopressor, Ventilator days, ICU length of stay and 28 day mortality) are now reported in Additional file 2: Table S1. The cohort of patients in this trial had a high incidence of respiratory failure. Nineteen patients had ARDS at entry as defined by the Berlin Definition with P_a_O_2_/F_i_O_2_ (PF) ratios of less than 300 and patchy airspace disease on chest imaging. Five patients had PF ratios above 300. Of the group with PF ratios above 300 only two were not intubated for ventilatory support. One patient in the group with PF ratios above 300 eventually fell below 300 and satisfied the Berlin Definition of ARDS.

**Table 1:** Baseline demographic data of septic patients treated or not treated with intravenous ascorbic acid

**Treatment**	**Gender**	**Age**	**APACHE II score**^**a**^	**SOFA score**^**b**^
Placebo	4 male 4 female	54 – 68 years	20.4 (15 – 29)	13.3 ± 2.9
Lo-AscA	5 male 3 female	30 – 70 years	20.4 (12 – 23)	10.1 ± 2.0
Hi-AscA	4 male 4 female	49 – 92 years	24.0 (12 – 33)	10.8 ± 4.4

^a^*APACHE* Acute Physiology and Chronic Health Evaluation, mean (range).

^b^*SOFA* Sequential Organ Failure Assessment, mean ± SE.

**Table 2:** Clinical data on patients with severe sepsis

**Underlying conditions**	**Source of sepsis**	**Organism**	**Renal failure?**	**Respiratory failure?**
Lung cancer	Pneumonia	Blood: E.coli, Strep bovis	no	yes
		Resp: E.coli		
Prodrome with nausea and vomiting for 7 days	Pneumonia	Blood: culture negative	no	yes
		Urine: Legionella antigen positive		
ETOH cirrhosis	Spontaneous bacterial peritonitis	Blood: culture negative	no	yes
Status/Post gastric bypass	Urinary tract infection	Blood: E. Coli	no	yes
Obstructive nephrolithiasis pyelonephritis	Urinary tract infection	Blood: E.Coli	no	yes
		Urine: E.Coli		
End stage renal disease	Catheter sepsis	Blood: MRSA	yes (prior to admission)	yes
Acute myelogenous leukemia (relapse)	Portacath sepsis	Blood: MRSA	no	yes
		Urine: Enterobacter		
Influenza	Pneumonia with coexistent Influenza A	Blood: Strep pneumonia	no	yes
		Resp: Influenza A		
Diabetes mellitus	Infected diabetic foot ulcer	Blood: Staph aureus	no	yes
Chronic kidney disease		Body Fluid: Staph aureus		
Gout		Resp: MRSA		
Head and neck cancer	Pneumonia	Blood: culture negative	no	yes
		Resp: culture negative		
Diabetes mellitus	Pneumonia and colitis	Blood: culture negative	yes (dialysis required)	yes
Congestive heart failure				
Gastrointestinal hemorrhage				
		Resp: MRSA		
Cellulitis	Pneumonia	Blood: Group a strep.	no	no
Hypercholesterolemia	Urinary tract infection			
Multiple myeloma	PneumoniaUrinary tract infection	Blood: Gram positive cocci	no	yes
		Urine: Proteus mirabilis		
Non-Hodgkins lymphoma	Pancreatitis	Resp: Aspergillus fumigatus	no	yes
Bone marrow transplant	Intra-abdominal sepsis	Blood: Gram negative rods, Gram positive cocci	no	yes
Bowel perforation				
Post allogeneic bone marrow transplant	Pneumonia	Blood: culture negative	no	yes
Chronic opiate use	Aspiration pneumonia	Blood: Strep. pneumonia	no	yes
Found obtunded		Resp: Strep. pneumonia, Candida glabrata		
Diabetes mellitus	Aspiration pneumonia	Resp: Gram negative rods, gram positive cocci	yes (prior to admission)	yes
End stage renal disease				
Chronic obstructive pulmonary disease	Pneumonia	Blood: culture negative	no	yes
		Resp: Acinetobacter, Stenotrophomonas maltiphilia		
Toxic epidermal necrolysis	Skin	Blood: MRSA	yes	yes
Acute renal failure				
Alcoholic cirrhosis	Subacute bacterial peritonitis	Blood: culture negative	no	no
Hepatorenal syndrome				
Chronic obstructive pulmonary disease	Pneumonia	Blood: Gram positive rods	no	yes
Severe ankylosing spondylitis	Urinary tract infection	Blood: Klebsiella pneumonia	no	yes
		Urine: Klebsiella pneumonia		
Severe aortic stenosis				
Hepatitis C cirrhosis	Health care acquired pneumonia	Blood: culture negative	yes (dialysis required)	yes
		Urine: Enterococcus		
Esophageal varicies				
Systemic mastocytosis	Pneumonia	Blood: culture negative	no	yes
Congestive heart failure		Resp: Budding yeast with pseudohyphae		

### Safety of intravenous ascorbic acid

Safety of ascorbic acid infusion in critically ill patients was a primary endpoint for this Phase I safety trial. During the 96-hour infusion period, no patients were withdrawn due to study-related adverse events (i.e., hypotension, tachycardia, hypernatremia, or nausea/vomiting). Infusions were halted in one septic patient (Hi-AscA) following infusion #14 (84 hours) for a ventricular arrhythmia later determined by Cardiology consultants to be electrical artifact. This patient is included in the analysis.

### Plasma ascorbic acid levels

**Figure 1:**
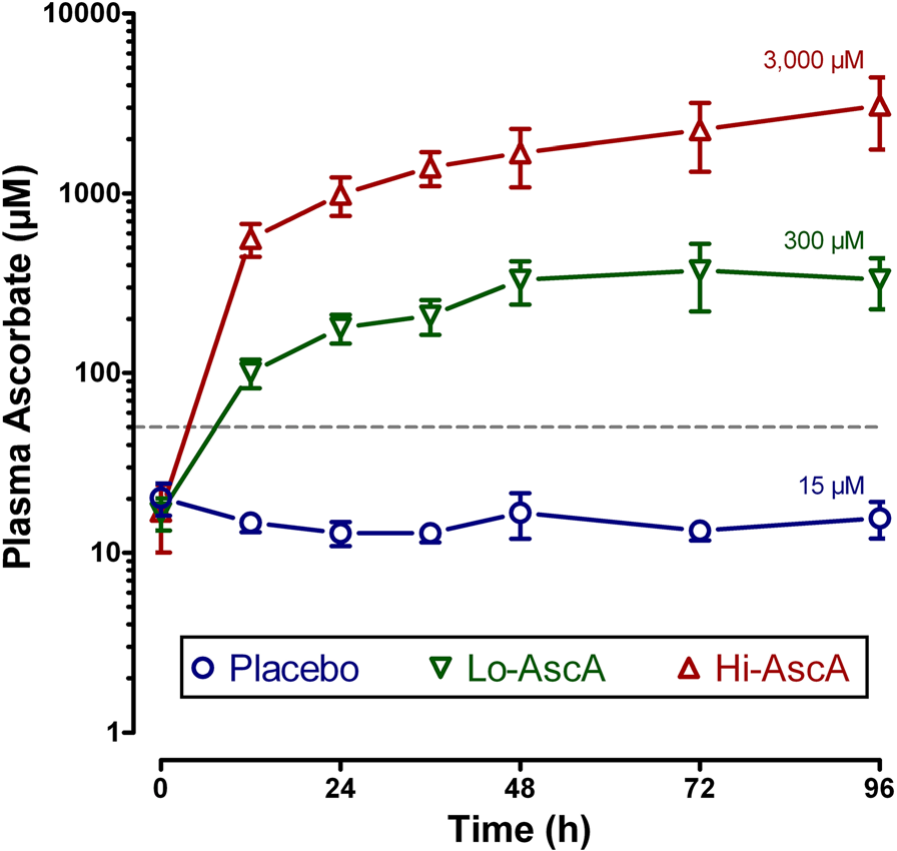
**Plasma ascorbic acid levels following intravenous infusion of ascorbic acid.** Plasma ascorbic acid levels were subnormal at entry (<50 μM, dotted line). Ascorbic acid levels rose rapidly in the two treatment groups and were significantly higher than placebo within twelve hours (Lo-AscA vs. placebo p < 0.005, Hi-AscA vs. placebo p < 0.0005) remaining consistently elevated for 96 hours. Ascorbic acid levels in the Hi-AscA group were significantly higher than the Lo-AscA group from the 12 hour point forward. These data show that an intermittent ascorbic acid infusion protocol (every 6 hours) produces sustained steady state levels in patients with severe sepsis. Placebo (О), Lo-AscA (▼), Hi-AscA (▲).

Plasma ascorbic acid levels in all septic patients at enrollment were subnormal (i.e., hyposcorbic) at 17.9 ± 2.4 μM (normal 50 – 70 μM) and were not significantly different at baseline (Figure 1). Ascorbic acid levels in the placebo group fell from 20.2 (11–45) μM at entry to 15.6 (7–27) μM on study day 4. Ascorbic acid levels increased 20-fold in the low dose treatment group from 16.7 (14–28) μM at baseline to 331 (110–806) μm on day 4. Ascorbic acid levels increased dramatically in Hi-AscA patients from 17.0 (11–50) μM at baseline to 3,082 (1,592 - 5,722) μm on day 4. Thus, ascorbic acid levels rose rapidly in the two treatment groups and were significantly higher than placebo within twelve hours (Lo-AscA vs. placebo p < 0.005, Hi-AscA vs. placebo p < 0.0005) remaining consistently elevated for the 96-hour infusion period. Furthermore, ascorbic acid levels in the Hi-AscA group were significantly higher (p < 0.005) than the Lo-AscA group from the 12 hour point forward reaching millimolar concentrations. These data confirm “hyposcorbic” levels present in untreated human sepsis and show that intermittent ascorbic acid infusion every 6 hours produces sustained steady-state plasma levels.

### Impact of ascorbic acid infusion on organ failure

**Figure 2:**
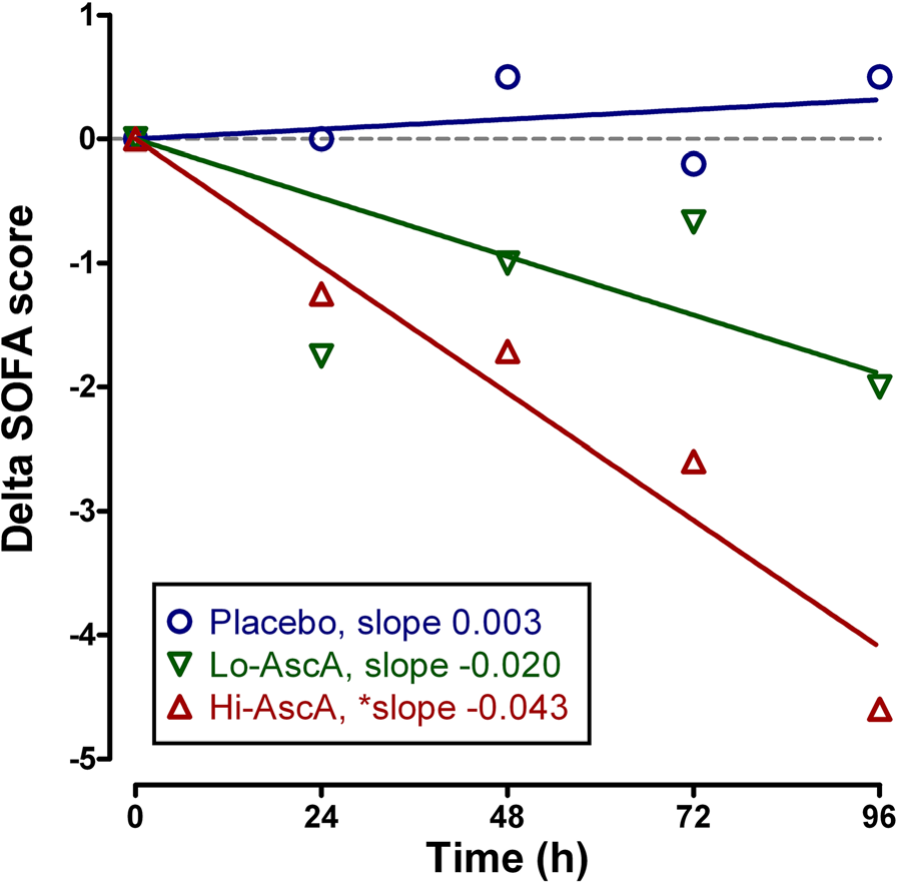
**Effect of ascorbic acid infusion on Sequential Organ Failure Assessment (SOFA) score (days 0–4).** Daily mean SOFA scores decreased over time with both doses of ascorbic acid infusion (p < 0.05 significantly non-zero) with the higher dose significantly less than placebo (Hi-AscA vs. placebo p < 0.01). Placebo (О), Lo-AscA (▼), Hi-AscA (▲).

SOFA scores at enrollment were: Placebo – 13.3 ± 2.9, Lo-AscA – 10.1 ± 2.0, and Hi-AscA 10.8 ± 4.4 and were not significantly different across groups. The components of the SOFA score are listed in Additional file 3: Table S2. Following normalization of the daily SOFA scores, patients treated with either dose of ascorbic acid exhibited descending SOFA scores over the 4-day study period (p < 0.05, slopes significantly non-zero). High dose ascorbic acid patients exhibited significantly faster declines in the regression slopes of delta daily total SOFA scores over time compared to placebo (-0.043 vs. 0.003, p < 0.01) (Figure 2). Placebo patients exhibited a gradual rise in SOFA scores. Though the cohort size is limited, these data suggest that ascorbic acid infusion significantly attenuates the systemic organ injury associated with sepsis.

### Impact of ascorbic acid infusion on biomarkers

**Figure 3:**
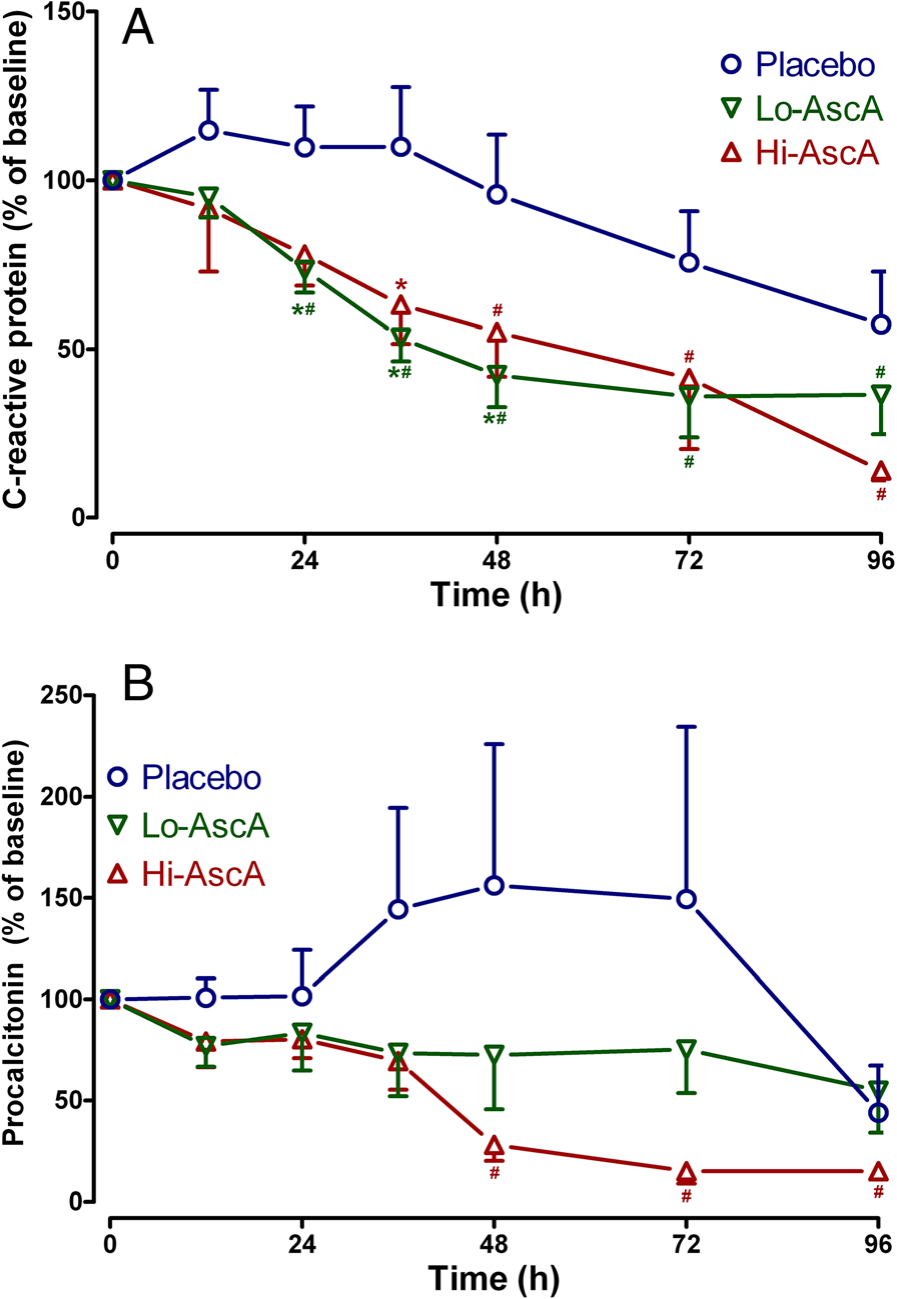
**Serum C-reactive protein (CRP) and procalcitonin levels in septic placebo controls and ascorbic acid infused patients. (A)** Both the Lo-AscA and the Hi-AscA dosages produced rapid reductions in serum CRP levels, becoming significantly lower than placebo (*p < 0.05 vs placebo) as early as 24 hours. Ascorbic acid infusion reduced CRP levels in both groups throughout the 4 study days (#p < 0.05 vs 0 hr). CRP levels in placebo patients slowly fell over the course of the 4 day study period. **(B)** Patients in the Lo-AscA and Hi-AscA groups exhibited reduced serum PCT levels beginning at 12 hours. Patients in the Hi-VitC group exhibited further significant reduction in serum PCT between 36 to 48 hours (#p < 0.05 vs 0 hr). Placebo patients exhibited a trend towards increased PCT levels which declined starting at 72 hours post onset of sepsis. Placebo (О), Lo-AscA (▼), Hi-AscA (▲).

**Figure 4:**
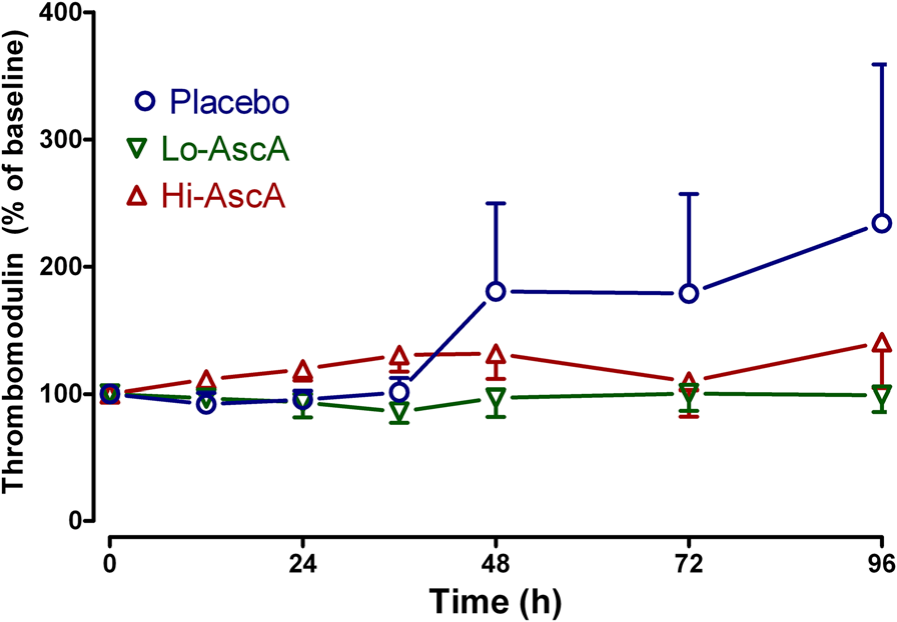
**Plasma thrombomodulin (TM) levels measured in septic placebo controls and ascorbic acid infused patients.** Plasma TM levels measured in the ascorbic acid infused patients exhibited no rise throughout the 4 days of study. Patients in the placebo group showed a trend towards increased plasma TM levels beginning at 36 hours, though it did not achieve statistical significance. Placebo (О), Lo-AscA (▼), Hi-AscA (▲).

Serum/plasma obtained from enrolled subjects were analyzed for three biomarkers: C-reactive protein (CRP), procalcitonin (PCT), and thrombomodulin (TM). CRP and PCT were quantified as surrogates for inflammation while TM was employed as a surrogate for endothelial injury. At enrollment, biomarker levels across the three groups were not significantly different. Serum CRP trended slowly down over the 96 hour period in the placebo group. Patients receiving ascorbic acid exhibited rapid reductions in CRP levels achieving significantly lower levels when compared to their own baseline and placebo by 24 hours (Figure 3A, p < 0.05). PCT levels trended higher in placebo-infused patients 24 hours following the onset of sepsis though not reaching statistical significance. Serum PCT levels in patients receiving high dose ascorbic acid declined, becoming significantly lower than baseline by 48 hours (Figure 3B, p < 0.05). PCT in patients receiving high dose ascorbic acid continued to decline over the 96-hour period. Plasma TM levels in patients randomized to placebo were not different from the ascorbic acid groups at baseline. Placebo patients began to trend upwards beyond 36 hours, remaining elevated when compared to ascorbic acid treated patients though the values were not statistically significant (Figure 4). Importantly ascorbic acid treated patients did not exhibit the upward trend in TM levels observed in placebo-infused patients. These results suggest that ascorbic acid infusion produces early reductions in proinflammatory mediators in patients with severe sepsis. The results further suggest that ascorbic acid infusion attenuates the evolution of endothelial injury characteristic of severe sepsis in humans.

## Discussion

This phase I trial focused on the safety of administering intravenous ascorbic acid to patients with severe sepsis. The intravenous route of administration was chosen in this trial in order to achieve high ascorbic acid plasma levels. Padayatty and colleagues showed that high-level ascorbic acid plasma concentrations could only be achieved by intravenous administration [15]. Prior human studies employing pharmacologic ascorbic acid dosing report no adverse events. Nathens et al. administered 1 gram of ascorbic acid every 8 hours for 28 days to surgically critically ill patients with no ill effects [16]. Tanaka et al. administered 66 mg/kg/hour for 24 hours to patients with 50% surface area burns with no adverse events [17]. Hoffer et al. intravenously administered up to 90 grams of ascorbic acid 3 times weekly to patients with advanced malignancy with no adverse events [30]. The dosing protocols we chose for this trial arose out of our preclinical work.

No patient in the low or high dose ascorbic acid treatment arms of this study suffered any identifiable adverse event. As noted above, the one instance in which ascorbic acid infusion was halted for a cardiac rhythm disturbance was determined to be artifact by the Division of Cardiology. Thus, a *pharmacologic* ascorbic acid treatment strategy in critically ill patients with severe sepsis appears to be safe.

Prior studies show that patients with severe sepsis exhibit significantly reduced plasma ascorbic acid levels upon admission to intensive care [31]. The mean initial plasma ascorbic acid level for all septic patients in this study was 17.9 ± 2.4 μM compared to normal human plasma levels of 50 – 70 μM (Figure 1). Prior studies [13,14,26], and the current study show *that subnormal plasma ascorbic acid levels are a predictable feature in patients with severe sepsis*. Importantly, Placebo patients exhibited no change in plasma ascorbic acid levels throughout the 4-day study period despite receiving full ICU standard of care practice for severe sepsis (Figure 1). Ascorbic acid depletion in sepsis results from ascorbic acid consumption by the reduction of plasma free iron, ascorbic acid consumption by the scavenging of aqueous free radicals (peroxyl radicals), and by the destruction of the oxidized form of ascorbic acid *dehydroascorbic acid*[14]. Sepsis further inhibits *intracellular* reduction of dehydroascorbic acid, producing acute intracellular ascorbic acid depletion. Sepsis-induced ascorbic acid destruction permits uncontrolled oxidant activity which amplifies tissue injury [14,32,33]. Ascorbic acid treated patients in this study exhibited rapid and sustained increases in plasma ascorbic acid levels using an intermittent every six hours administration protocol (Figure 1).

SOFA scores are robust indicators of mortality during critical illness. SOFA score increases during the first 48 hours of ICU care predict a mortality rate of at least 50% [26]. In this study, the extent of organ failure accompanying patients with severe sepsis was high with an average SOFA score for all patients equal to 11.4 ± 3 confirming that multiple organ failure was present at enrollment. Given that the mean plasma ascorbic acid levels on admission were subnormal (17.9 ± 2.4 μM), a mean initial SOFA score of 11.4 ± 3 in patients with severe sepsis was not surprising. This study is in agreement with other studies which show that plasma ascorbic acid levels in severe sepsis correlate *inversely* with the incidence of multiple organ failure [13]. We showed that the addition of ascorbic acid to standard of care practice (i.e., fluid resuscitation, antibiotics, vasopressor medication) for patients with severe sepsis significantly reduced organ injury. Ascorbic acid treated patients exhibited prompt and sustained reductions in SOFA scores during the 4-day treatment regimen unlike placebo controls where SOFA scores slowly increased over time. SOFA score reduction was most remarkable in patients receiving the high dose ascorbic acid infusion (Figure 2).

C-reactive protein (CRP) [19] and procalcitonin (PCT) [20] levels are known to correlate with the overall extent of infection and higher levels of both have both been linked to higher incidences of organ injury and death in the critically ill. CRP in circulation has a short half-life of approximately 19 hours. Thus, the kinetics of CRP make it a useful monitor for tracking the inflammatory response produced by infection, and the response to antibiotic treatment. Lobo et al. reported that patients with CRP levels greater than 10 mg/dL at ICU admission exhibited significantly higher rates of multiple organ failure as well as higher mortality rates [34]. A decrease in CRP levels in Lobo’s study after 48 hours was associated with a mortality rate of only 15.4%, while a persistently high CRP level was associated with a mortality rate of 60.9%. Both low and high dose ascorbic acid infusion in this trial promptly reduced serum CRP levels in septic patients (Figure 3A). Thus, the findings in this study support the findings of Lobo et al. with descending CRP levels being associated with lower mortality rate and reduced levels or organ failure. Jensen and colleagues found that high maximal procalcitonin levels were an early independent predictor of all-cause mortality in a 90-day follow-up period after intensive care unit admission [20]. Karlsson and colleagues [21] showed that mortality in patients with severe sepsis was lower in those patients in whom procalcitonin concentrations fell by more than 50% at 72 hours with respect to initial values. Infusion of ascorbic acid into patients with severe sepsis in this study reduced serum procalcitonin levels by greater than 50% (Figure 3B). Thrombomodulin is an endothelial cell bound molecule that captures thrombin holding it adjacent to protein C bound to its receptor (endothelial protein C receptor). Elevated soluble TM in the circulation indicates endothelial cell injury [35]. Lin et al. reported that increased TM levels correlated with the extent of organ failure and mortality in patients with sepsis [36]. In the current study, thrombomodulin levels in patients randomized to placebo began to rise at approximately 36 hours into the study period, indicating sepsis-induced endothelial injury (Figure 4). Patients randomized to receive either dose of ascorbic acid exhibited no subsequent rise in plasma thrombomodulin. Though our patient numbers were small, these early results suggest that intravenous ascorbic acid acts to attenuate the proinflammatory state of sepsis and perhaps attenuates the development of endothelial injury.

On the basis of this study and our prior preclinical studies, we speculate as to the pleiotropic mechanisms by which ascorbic acid would be beneficial in sepsis. Ascorbic acid is rapidly taken up by endothelial cells in millimolar quantities where it scavenges reactive oxygen species and increases endothelial nitric oxide synthase-derived nitric oxide by restoring tetrahydrobiopterin content, thus, increasing bioavailable nitric oxide. As we and others have shown in basic investigations [10,11,37], by *inhibiting* NFκB activation, ascorbic acid could potentially attenuate the “cytokine storm” that arises due to NFκB driven genes known to be activated in sepsis. Septic ascorbic acid-deficient neutrophils fail to undergo normal apoptosis. Rather, they undergo necrosis thereby releasing hydrolytic enzymes in tissue beds, thus contributing to organ injury. We speculate that intravenous ascorbic acid acts to restore neutrophil ascorbic acid levels. Repletion of ascorbic acid in this way allows for normal apoptosis, thus, preventing the release of organ damaging hydrolytic enzymes. A multitude of biological mechanisms are active in patients with sepsis and they promote multiple organ injury and death.

Tens of thousands of lives are lost across the world annually due to severe sepsis [1,38-40]. Multiple treatment trials have failed to measurably improve outcomes. The majority of trials have singly eliminated certain proinflammatory mediators which research has suggested promotes tissue damage. The single mediator approach has largely been unsuccessful. The results from this small phase I safety trial suggest that administering ascorbic acid in pharmacological dosages to critically ill patients with sepsis is safe and that it may provide *adjunctive therapy* in the treatment of severe sepsis. A larger phase II proof-of-concept trial is needed.

## Conclusions

This phase I trial shows that aggressive repletion of plasma ascorbic acid levels in patients with severe sepsis is safe. This early work in septic patients suggests that pharmacologic ascorbic acid repletion reduces the extent of multiple organ failure and attenuates circulating injury biomarker levels.

## Abbreviations

AscA: Ascorbic acid; CRP: C-reactive protein; PCT: Procalcitonin; SOFA: Sequential organ failure assessment; TM: Thrombomodulin.

## Competing interests

The authors declare that they have no competing interests.

## Authors’ contributions

AAF, RN, BJF, AAS: Hypothesis/delineation. AAF, AAS, DF, RS, SK, CD: Study design. AAF, AAS, RN, BJF, DF, CAF, TLL, CD, SG, EM, DFB, MRICU Nursing: Acquisition of data/analysis. AAF, RN, BJF, DF, RS: Interpretation of data/writing the article. AAF, RN, AAS, and BJF conceived, designed, or planned the study, interpreted the results, and wrote sections of the initial draft. DF, CAF, and TLL designed, validated and performed the plasma ascorbic acid HPLC analysis. SK, CD aided in study design and data collection. RS helped design the study and wrote sections of the initial draft. SG supervised biomarker analysis and interpretation of results. EM and DFB provided substantial review and suggestions of the initial draft. All authors read and approved the final manuscript.

## Supplementary Material

Additional file 1 **Patient Flow Diagram.** Flow diagram of the progress through the phases of the safety trial (enrollment, allocation, follow-up, and analysis). Click here for file

Additional file 2: Table S1 Secondary outcomes of septic patients treated or not treated with intravenous ascorbic acid. Includes days on vasopressor, ventilator free days, ICU length of stay, and 28-day mortality. Click here for file

Additional file 3: Table S2 Components of the Sequential Organ Failure Assessment (SOFA) scoring system. Describes the clinical parameters of the scoring system. Click here for file
