# Supplementary figures and images for: Phase I safety trial of intravenous ascorbic acid in patients with severe sepsis
Source: J Transl Med. 2014 Jan 31;12:32. doi: 10.1186/1479-5876-12-32 (PMC3937164; doi:10.1186/1479-5876-12-32)

## Patient Flow Diagram

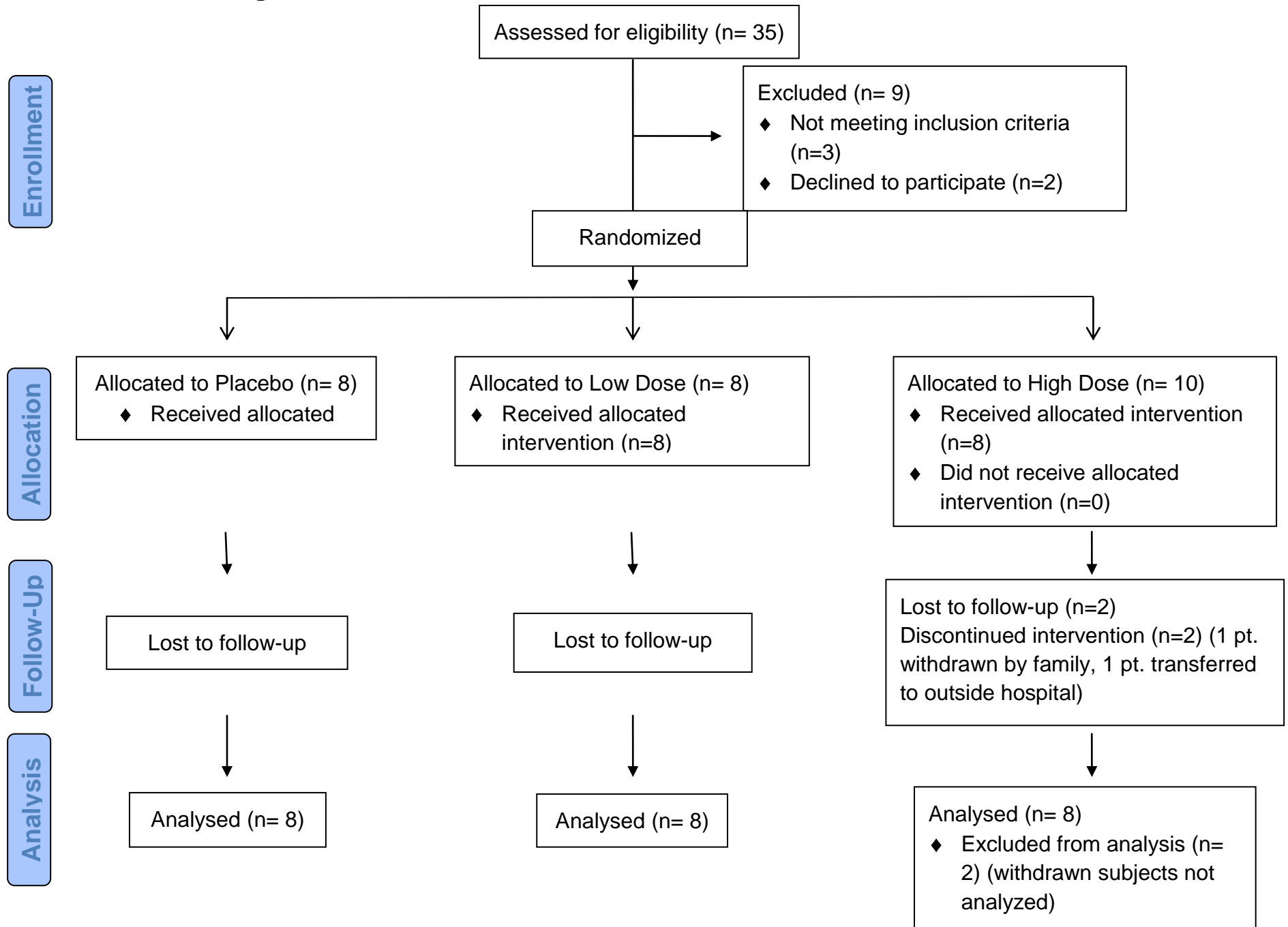

Supplement: Additional file 1 — Patient Flow Diagram. Flow diagram of the progress through the phases of the safety trial (enrollment, allocation, follow-up, and analysis). [file 1479-5876-12-32-S1.pdf]
