# Supplementary material for: Phase I safety trial of intravenous ascorbic acid in patients with severe sepsis
Source: J Transl Med. 2014 Jan 31;12:32. doi: 10.1186/1479-5876-12-32 (PMC3937164; doi:10.1186/1479-5876-12-32)
Supplement: Additional file 2: Table S1 — Secondary outcomes of septic patients treated or not treated with intravenous ascorbic acid. Includes days on vasopressor, ventilator free days, ICU length of stay, and 28-day mortality. [file 1479-5876-12-32-S2.pdf]

Supplementary Table 1. Secondary outcomes of septic patients treated or not treated with intravenous ascorbic acid

Treatment Group	Days on Vasopressor	Ventilator Free Days	ICU Length of Stay	28-Day Mortality
Placebo	3.9 (1 – 10)	7.6 (0 – 23)	11.0 (2 – 25)	62.5%
Lo-AscA	2.1 (1 – 6)	8.4 (0 – 22)	8.1 (1 – 19)	38.1%
Hi-AscA	3.6 (2 – 8)	4.8 (0 – 19)	9.1 (2 – 25)	50.6%

Data reported as mean (range)
