# Supplementary material for: Phase I safety trial of intravenous ascorbic acid in patients with severe sepsis
Source: J Transl Med. 2014 Jan 31;12:32. doi: 10.1186/1479-5876-12-32 (PMC3937164; doi:10.1186/1479-5876-12-32)
Supplement: Additional file 3: Table S2 — Components of the Sequential Organ Failure Assessment (SOFA) scoring system. Describes the clinical parameters of the scoring system. [file 1479-5876-12-32-S3.pdf]

Supplementary Table 2. Components of the Sequential Organ Failure Assessment (SOFA) scoring system

	Respiratory	Neurological	Cardiovascular	Hepatic	Coagulation	Renal
**Organ Failure Score**	PaO_2/FiO_2 (mmHg)	Glasgow Coma Score	Mean Arterial Pressure If hypotensive then vasopressor requirement	Bilirubin (mg/dL)	Platelets ($x10^3/\mu l$)	Creatinine (mg/dL)
**1**	< 400	13 - 14	MAP < 70 mmHg	1.2 - 1.9	< 150	1.2 - 1.9
**2**	< 300	10 - 12	Dopamine ≤ 5 µg/kg/min	2.0 - 5.9	< 150	2.0 - 3.4
**3**	< 200 mechanically ventilated	6 - 9	Norepi ≤ 0.1 µg/kg/min	6.0 - 11.9	< 50	3.5 - 4.9
**4**	< 100 mechanically ventilated	< 6	Norepi > 0.1 µg/kg/min	> 12	< 20	> 5

SOFA score equals the sum of six organ failure scores
